# Multi-cohort analysis reveals immune subtypes and predictive biomarkers in tuberculosis

**DOI:** 10.1038/s41598-024-63365-5

**Published:** 2024-06-10

**Authors:** Ling Li, Tao Wang, Zhi Chen, Jianqin Liang, Hong Ding

**Affiliations:** The Eighth Medical Center of the PLA General Hospital, Beijing, 100091 People’s Republic of China

**Keywords:** Tuberculosis, Immune microenvironment, PTB, Subtypes, Neural network model, Single-cell, Computational platforms and environments, Data acquisition, Data mining

## Abstract

Tuberculosis (TB) remains a significant global health threat, necessitating effective strategies for diagnosis, prognosis, and treatment. This study employs a multi-cohort analysis approach to unravel the immune microenvironment of TB and delineate distinct subtypes within pulmonary TB (PTB) patients. Leveraging functional gene expression signatures (Fges), we identified three PTB subtypes (C1, C2, and C3) characterized by differential immune-inflammatory activity. These subtypes exhibited unique molecular features, functional disparities, and cell infiltration patterns, suggesting varying disease trajectories and treatment responses. A neural network model was developed to predict PTB progression based on a set of biomarker genes, achieving promising accuracy. Notably, despite both genders being affected by PTB, females exhibited a relatively higher risk of deterioration. Additionally, single-cell analysis provided insights into enhanced major histocompatibility complex (MHC) signaling in the rapid clearance of early pathogens in the C3 subgroup. This comprehensive approach offers valuable insights into PTB pathogenesis, facilitating personalized treatment strategies and precision medicine interventions.

## Introduction

Tuberculosis (TB) stands as a major global infectious disease, posing significant challenges to both human health and healthcare systems worldwide^1,2^. Currently, a primary challenge lies in identifying latent tuberculosis infection (LTBI), given that 90% of infected individuals exhibit no overt symptoms or signs, yet remain at risk of developing active tuberculosis in the future. The TB infection cycle entails numerous critical events, spanning from initial infection to the establishment of latency, involving a series of intricate processes within host cells. Mycobacterium tuberculosis engages in a protracted battle with the host, ensuring its survival by modulating metabolic environments and immune responses^3–5^. In this context, comprehending the clinical classification of TB and the behavior of the pathogen across different classifications is pivotal for predicting disease progression and determining the most appropriate treatment approach.

Generally, TB is classified based on several criteria including clinical features, pathological presentations, Mycobacterium tuberculosis drug resistance, and molecular epidemiology^6^. Clinically, it can be divided into primary and secondary forms, while pathologically, it may manifest as caseous, infiltrative, or fibrotic TB. Drug resistance classification includes identifying drug-resistant tuberculosis strains. Molecular epidemiology techniques are also employed for strain typing^7,8^. These classification methods collectively offer insights into the development, transmission, and treatment status of TB, guiding clinical treatment and disease control efforts. Although there's no definitive transcriptomics-based classification system yet, some studies may categorize TB into subtypes based on patient immune responses, pathogen metabolism, and treatment responses^9,10^. Revealing these subtypes enhances understanding of TB progression and treatment response, facilitating personalized treatment and precision medicine. However, these classification systems are still in the research phase and haven't seen widespread adoption in clinical practice.

The immune environment plays a critical role in the development of TB, influencing how the disease progresses^11^. After infection, the tuberculosis bacteria cause lung inflammation and immune cell buildup, creating an environment that promotes inflammation^6,12,13^. However, the bacteria also manipulate the immune response, making it harder for the body to clear them. This can lead to lung damage and recurring infections in some cases^4,9^. Understanding these changes in the immune system is crucial for finding better treatment strategies. Currently, studying the different types of immune environments in TB has some challenges. There isn't a clear way to define or classify these types, making it hard to compare research findings^5,14,15^. Also, most studies focus on the levels of different immune cells and proteins, without considering how these cells interact or how signaling pathways are regulated. Plus, because many factors can affect a TB patient's immune system, it's tough to create a complete model of immune environment types.

Using functional gene expression signatures (Fges) provided by Bagaev et al.^16^, which represent the main functional components of the TB microenvironment, we investigated how these gene sets are active in TB patients and how they relate to prognosis. By analyzing the diversity triggered by Fges enrichment scores, we focused on pulmonary tuberculosis (PTB) patients and successfully divided them into three distinct subtypes. This allowed us to explore the molecular characteristics and functional differences within the microenvironment, uncovering key molecular mechanisms behind the prognostic disparities among PTB subtypes. To ensure applicability to other PTB patient groups, we developed a dependable classification model using neural networks. Additionally, in conjunction with drug sensitivity analysis, we offered potential clinical guidance for treatment.

## Materials and methods

### Data source and preprocessing

This study presents an in-depth analysis of transcriptomic data obtained from the "curatedTBData" package, accessible at https://github.com/wejlab/curatedTBData. The datasets analyzed in this study comprise 49 transcriptomic studies, which include gene expression profiles from patients diagnosed with TB as well as individuals with various other clinical conditions. To ensure the reliability and effectiveness of the analysis, stringent criteria were applied to exclude datasets with fewer than 15,000 genes or fewer than 10 samples.

Through this rigorous screening process, 31 high-quality datasets were identified, including GSE101705 (n = 44), GSE107104 (n = 33), GSE112104 (n = 51), GSE19435 (n = 33), GSE19439 (n = 42), GSE19442 (n = 51), GSE19443 (n = 44), GSE19444 (n = 54), GSE22098 (n = 274), GSE25534 (n = 102), GSE28623 (n = 108), GSE29536 (n = 15), GSE34608 (n = 44), GSE37250 (n = 537), GSE39939 (n = 157), GSE39940 (n = 334), GSE40553 (n = 204), GSE41055 (n = 27), GSE42825 (n = 42), GSE42826 (n = 102), GSE42830 (n = 95), GSE42832 (n = 90), GSE50834 (n = 44), GSE54992 (n = 39), GSE56153 (n = 71), GSE62147 (n = 52), GSE62525 (n = 42), GSE69581 (n = 50), GSE73408 (n = 109), GSE83456 (n = 202), and GSE83892 (n = 116). Each dataset underwent standardization within the "*curatedTBData*" package to ensure uniformity and comparability across studies. For further details on expression values and associated metadata, please refer to Supplementary Table S1.

### Calculation of enrichment score (ES) and signature score

To calculate the ESs of Fges gene sets in each TB sample (Table S2), the "gsva" function from the *GSVA* package (version 1.50.0)^17^ was employed. Then normalization was conducted to ensure that the ESs are non-negative, using the following formula.

$${\varvec{S}}=\frac{{\varvec{E}}{\varvec{S}}-\text{min}({\varvec{E}}{\varvec{S}})}{\text{max}\left({\varvec{E}}{\varvec{S}}\right)-\text{min}({\varvec{E}}{\varvec{S}})}$$

***ES*** is a matrix of ESs, where rows represent Fges sets and columns represent TB samples. ***S*** denotes the normalized ES (NES), with a range from 0 to 1.

For the signature scores of a gene list mentioned in the present study, the Seurat tool's *AddModuleScore* function^18^ was utilized.

### Quantifying heterogeneity with Jensen-Shannon divergence

To quantify the heterogeneity of Fges-related ESs in the TB cohorts, Jensen-Shannon divergence (JSD) is employed as a metric for assessing the similarity between two probability distributions. The formula is as follows:

$$JSD\left(P,Q\right)=\frac{1}{2}\left({D}_{KL}\left(P||M\right)+{D}_{KL}\left(Q||M\right)\right)$$

where $P=\frac{{{\varvec{S}}}_{i.}}{\sum_{j=1}^{n}{{\varvec{S}}}_{ij}}$, $Q={\left[\frac{1}{n}\right]}_{n}$, and M = $\frac{1}{2}\left(P+Q\right)$. ***S*** is a matrix of NES mentioned previously, where rows represent Fges sets and columns represent TB samples.

### Inference of PTB patient subtypes

Immune-related pathways (C7) were retrieved from the MsigDB database, consisting of 5214 functional gene sets representing cell states and immune system perturbations. GSVA was then applied to calculate the ESs of these pathways in PTB patients (n = 1728) from 31 TB datasets. Subsequently, the correlation between these pathway enrichment scores and the activity scores of cytokine signaling-related gene sets was computed, with only those exhibiting correlation coefficients greater than 0.5 being retained. Utilizing the resulting matrix of pathway enrichment scores in PTB patients, hierarchical clustering was performed on the TB patients using “Euclidean” distance and the “complete” linkage method. This analysis revealed three distinct subtypes, labeled as C1 (n = 432), C2 (n = 560), and C3 (n = 736).

### Estimation of cellular compositions of PTB subgroups

To investigate the differences in cell type composition among various PTB subtypes, deconvolution analysis was separately performed on PTB patients using xCell^19^ and CIBERSORT^20^ to estimate their cellular compositions. xCell includes 64 default cell types, while CIBERSORT utilizes the LM22 base matrix, encompassing 22 crucial immune cell types. Default parameters were maintained for both tools during the analysis.

### Differentially expressed genes (DEGs) and functional enrichment analysis

Utilizing the *FindMarkers* function from the “Seurat” package (version 4.3.0)^21^, Wilcoxon Rank Sum tests were employed to identify significantly differentially expressed genes (DEGs) between each PTB subgroup and the remaining subgroups. Bonferroni correction was applied to the *p*-values for multiple testing, and genes with corrected p-values less than or equal to 0.05 were considered significant DEGs. These identified genes were subsequently used for Gene Ontology (GO) and Kyoto Encyclopedia of Genes and Genomes (KEGG) enrichment tests using the R package *ClusterProfiler* (version 4.0.5)^22^.

### Constructing biomarkers for PTB patient progression and predicting subgroups with neural network models

To develop biomarkers for predicting the disease progression status of PTB patients, a neural network model was employed with the following steps: First, a set of 1829 feature genes exhibiting significantly higher expression in C3 compared to C1 and C2 was identified. The average expression of these genes across different PTB subtypes was calculated, resulting in an expression profile of 1829 × 3. Using Spearman correlation analysis, the alignment of each gene's expression trends with the cytokine signaling activity trends from C1 to C3 was assessed. Genes with a correlation of 1 were retained, yielding 43 differential expression genes. Subsequently, a neural network model was constructed using the expression profile of these 43 feature genes as input (1728 PTB samples × 43 feature genes). The model comprised two hidden layers: one with 64 neurons and the other with 32 neurons, utilizing *ReLU* activation functions. To obtain predicted scores within the range of [− 1, 1], the tanh activation function was applied and passed to the output layer. Finally, the mean squared error (MSE) is utilized as the loss function.

Next, to further predict various subgroups among PTB patients, modifications were made to the neural network model used for predicting disease progression. These adjustments involved: randomly dividing 1728 PTB patient samples into training and independent testing sets with a 7:3 ratio; defining the feature genes in the input layer as all significantly differentially expressed genes obtained from C1, C2, and C3 (2314 genes); introducing a Dropout strategy (rate = 0.2) in the hidden layer to mitigate model overfitting and improve generalization; configuring three neurons in the output layer to represent three distinct PTB subtypes; using the *softmax* function as the activation function. Finally, the cross-entropy loss function was employed.

### Deriving PTB insights from single-cell RNA-seq data

Given the scarcity of extensive single-cell data of PTB patients, HABERMANN et al.^23^ have provided a breakthrough by sharing single-cell data for 10 non-fibrotic control and 20 pulmonary fibrosis (PF) lungs (accession number: GSE135893). Despite the distinct nature of PF and PTB, they may share certain biological characteristics, such as irregular changes in lung cells. Hence, delving into PF data may yield valuable insights into PTB subtypes, particularly in terms of identifying and predicting cell subtypes. To accomplish this, a pre-trained neural network model was employed to forecast potential subtypes in these PF patients. The process involves calculating the average expression of each gene across all cells in each single-cell PF sample, generating a vector of average gene expression for each sample. These average expression signals from all single-cell samples are then amalgamated into a matrix, with rows representing genes and columns representing samples, depicting the average expression signals of genes in samples. Subsequently, the pre-trained neural network model is utilized to predict the classification of each sample. The final outcomes reveal: C1 (n = 13); C2 (n = 6); C3 (n = 11).

### Cellchat for cell–cell communication analysis

For inferred potential ligand-receptor (L-R) pairs for regulating the differences among PTB subgroups, CellChat (version 1.6.1)^24^ was employed to identify cell–cell interactions based on the expression of known L-R pairs in different cell types. The input for CellChat comprised gene expression data of cells along with their assigned cell types. Initially, overexpressed ligands or receptors within specific cell groups were identified, and the gene expression data was projected onto a protein–protein interaction network. Subsequently, CellChat facilitated the inference of biologically significant cell–cell communication by assigning a probability value to each interaction and conducting a permutation test. Finally, the resulting communication networks were visualized using a circle plot, and the signaling pathways were visualized using a bubble plot.

### Statistical analysis

Pearson correlations were utilized to evaluate concordance between groups. Various standard statistical tests, such as Student's *t*-test, and Wilcoxon rank-sum test were employed to analyze both clinical and expression data within the study. Principal Component Analysis (PCA) is utilized to illustrate the distribution of samples from three distinct subtypes of PTB. All statistical analyses were performed using R version 4.3.2.

## Results

### Insights into tuberculosis immune microenvironment from multi-cohort analysis

**Figure 1:**
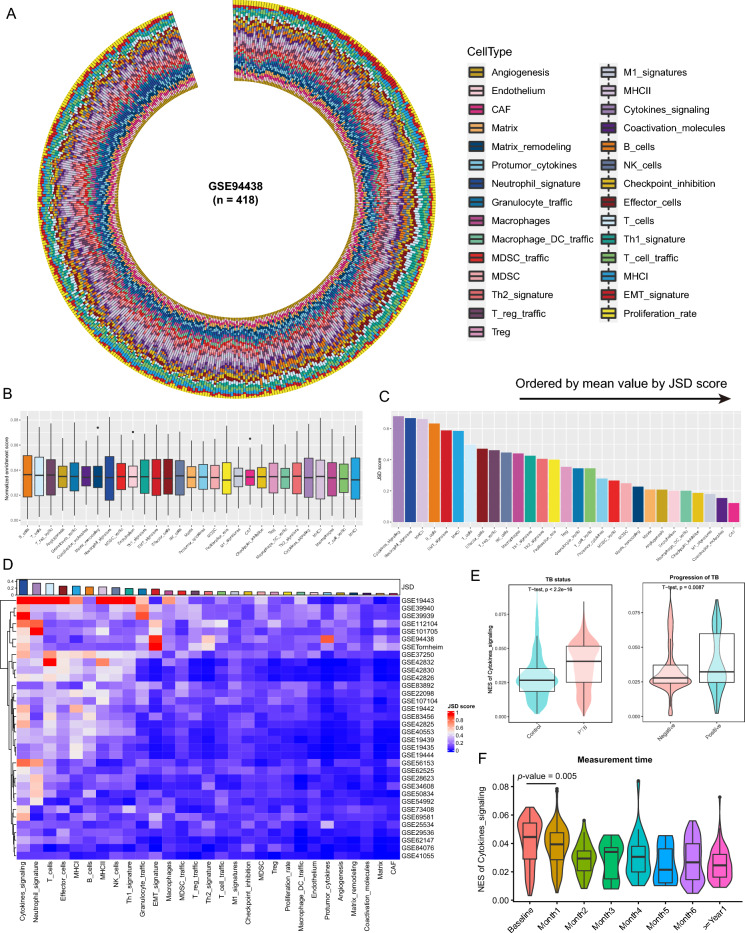
Exploring enrichment scores of Fges-related gene sets in tuberculosis cohorts. (**A**) Circular plot illustrating the distribution of enrichment scores (ESs) for 29 Fges-related gene sets in the GSE94438 TB cohort (n = 498). Each bar represents an individual sample, and the color code indicates varying ESs of gene sets. (**B**) Boxplot presenting the scaled distribution of ESs for the 29 Fges-related gene sets (referred to as Fges). The median value is depicted by the black line within the box, while outliers are indicated by black points outside the box. (**C**) Bar plot displaying the Jessen-Shannon divergence (JSD) scores for the Fges, arranged in descending order from left to right. (**D**) Heatmap depicting the ESs of the Fges across 34 TB cohorts. The annotation bar plot shows the JSD scores of the Fges. Rows in the heatmap are clustered using hierarchical clustering. (**E**) Violin plots illustrating the distribution of cytokine scores between different groups: normal controls vs. TB patients (left) and TB patients with negative vs. positive progression (right), with *p*-values obtained through t-tests. (**F**) Violin plot presenting the distribution of cytokine scores along the progression time of PTB exposure, from baseline to exposure greater than 1 year. *P*-value was obtained through t-test.

To delve into the immune microenvironment of TB, a meticulous selection of 29 functional gene sets (Fges) was made, encapsulating key functional elements of immune, stromal, and other cellular constituents. These gene sets were curated based on previous research endeavors^24^ (Tables S2). Subsequently, employing the Gene Set Variation Analysis (GSVA) method (method = "gsva")^17^, the enrichment scores (ESs) of Fges among a cohort of TB patients (n = 498) sourced from the GSE94438 dataset were scrutinized (see “Materials and methods”; Table S1). A prevailing uniformity in the pattern of Fges-associated ESs across TB patients was unearthed, hinting at a degree of consistency in immune, stromal, and other cellular responses within this population (Fig. 1A and B). Such consistency may suggest a relative stability in the interactions or regulatory mechanisms among diverse cell types within this specific TB patient cohort.

Notwithstanding the overall semblance in the ESs of these distinctive gene sets among TB patients, a certain level of heterogeneity was discerned (Fig. 1A and B). To quantify this heterogeneity, Jensen-Shannon divergence (JSD) scores were introduced, with higher scores indicating greater heterogeneity (see “Materials and methods”). Findings revealed that cytokine signaling exhibited the highest average heterogeneity level, whereas fibroblasts (CAF) showcased the lowest (Fig. 1C). The pronounced heterogeneity in cytokine signaling may imply significant inter-patient disparities in the immune system, reflective of the heterogeneous nature of immune responses across different patients. Conversely, the fibroblast-related gene set, renowned for their supportive and reparative roles in lung tissue, exhibited low heterogeneity, suggesting relative stability in the function and characteristics of these cells in TB patients. To validate these findings further, an additional analysis was conducted on data from 34 additional TB patient cohorts (Table S2). Similarly, the analysis illuminated the highest heterogeneity (JSD) in cytokine signaling among these cohorts, and CAF with the lowest (Fig. 1D). This underscores the pivotal role of cytokine-related gene sets in the onset and progression of TB. Consequently, an exploration of the disparities in cytokine signaling among distinct TB patient statuses was embarked upon. Both the normal control group and PTB cohorts showcased a higher ES in PTB relative to the normal control group, indicating significant distinctions (Fig. 1E). In the progression of TB patients, cytokine scores of TB-positive patients were notably higher than those of TB-negative patients (Fig. 1E). Notably, fluctuations in the time elapsed from PTB onset revealed that PTB patients exhibited peak cytokine enrichment scores at the exposure stages (baseline), gradually waning as the temporal distance from PTB onset increased (Fig. 1F). This suggests that exposure-stage PTB patients exhibit heightened immune responses, potentially serving as a pivotal indicator of PTB patient progression.

### Cytokine signaling heterogeneity reveals distinct subtypes in pulmonary tuberculosis

**Figure 2:**
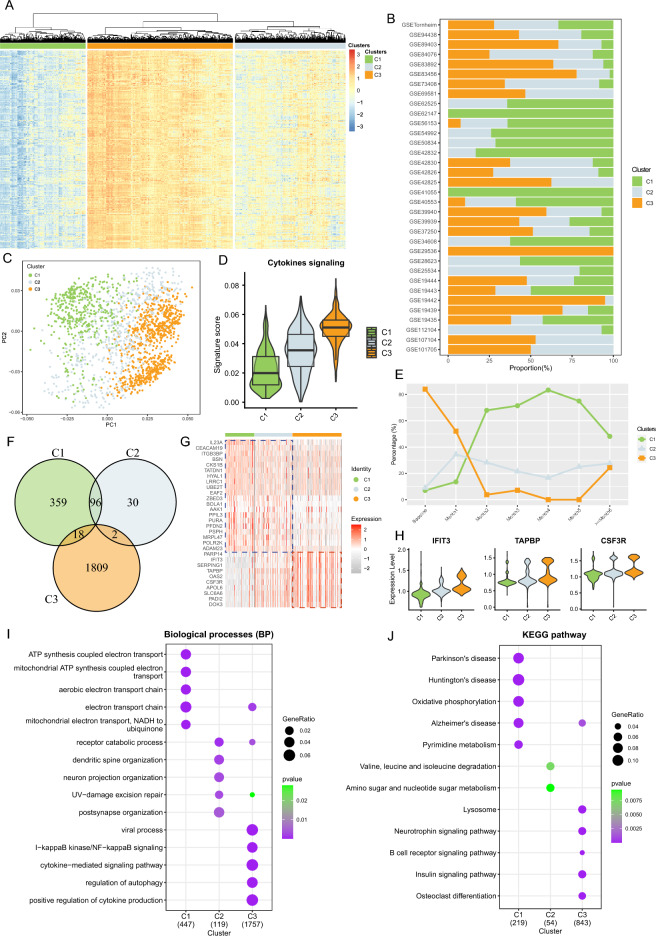
Fges enrichment scores reveal three subgroups within the PTB cohorts. (**A**) Hierarchical clustering heatmap illustrating clusters formed based on the ES of 29 Fges across 34 TB cohorts. (**B**) Stacked bar plot depicting the percentage distribution of three clusters within each TB cohort, with clusters identified by color codes. (**C**) Scatter plot displaying the distribution of PTB samples using principal component analysis. Each dot represents an individual sample, and clusters are differentiated by color codes. (**D**) Violin combined with box plot showcasing the signature score of cytokine gene sets across different PTB subgroups. (**E**) Line graph with scatter plot illustrating the percentage of samples in each time period from PTB exposure to greater than months, with clusters distinguished by color. (**F**) Venn diagram demonstrating the gene overlap among the three PTB subgroups. (**G**) Heatmap revealing the expression of selected over-expressed genes in PTB patients. The column annotation bar indicates the cluster to which each sample belongs. (**H**) Violin plot illustrating the expression of cytokine-related genes within the three PTB subgroups. (**I**) Bubble plot displaying enriched gene ontologies among the three PTB subgroups. Color change indicates the significance of enrichment, and the size of the points represents the number of genes in each GO term. (**J**) Bubble plot depicting enriched KEGG pathways among the three PTB subgroups. Color variation signifies the significance of enrichment, and the size of the points indicates the number of genes in each KEGG pathway.

Within TB patients, there's a notable heterogeneity in cytokine signaling, indicating potential variations in TB clinical subtypes. Given the predominance of PTB within TB cases relative to other types, encompassing LTBI, EPTB (Extrapulmonary tuberculosis), OD (Organ-specific tuberculosis), and Subclinical (Subclinical tuberculosis), our focus was on uncovering potential subtypes within PTB patients (Table 1). To delve into this, immune-related pathways (C7) from the MsigDB database were selected and GSVA tool was employed to compute their ESs across 31 TB datasets specifically in PTB patients. Subsequently, their correlations with the ESs of cytokine signaling-related gene sets were examined. Retaining the pathways from C7 with Pearson correlation coefficients surpassing 0.5, we proceeded to hierarchically cluster PTB patients from the TB cohorts based on the ESs of these pathways, which unveiled three distinct subgroups denoted as C1, C2, and C3 (Fig. 2A; Table S3). Notably, C1 exhibited the lowest immune-inflammatory activity, in contrast to C3, which displayed the highest. Figure 2B demonstrated the distribution of these subgroups across various PTB cohorts (Fig. 2B). Additionally, by further validating these subgroup distributions, principal components analysis (PCA) underscored their disparities (Fig. 2C). The activity levels of cytokines in the three distinct subgroups corresponded to the immunoreactivity revealed by hierarchical clustering, with C3 being the highest and C1 the lowest (Fig. 2D). Furthermore, the associations between different subgroups and stages of disease progression in PTB patients were examined, with C3 prevalent during the outbreak phase, while C1 showed gradual increases during the recovery phase (Fig. 2E). Over time, these discrepancies diminished, indicating evolving inherent variability among subtypes during disease progression.

**Table 1:** Clinical characteristics of various tuberculosis disease states.

Characteristic	Description	Control	PTB	LTBI	EPTB	OD	Subclinical
Symptoms	Presence of symptoms such as persistent cough, fever, night sweats, weight loss, and fatigue	Absent	Present	Absent	Variable	Variable	Variable
Tuberculin skin test (TST) or interferon-gamma release assay (IGRA)	Positive result indicating exposure to tuberculosis bacteria	Negative	Positive	Positive	Positive	Positive	Positive
Chest X-ray	Abnormal findings such as infiltrates, cavities, or consolidations	Normal	Abnormal	Normal	Abnormal	Abnormal	Abnormal
Bacteriological confirmation	Presence of acid-fast bacilli in sputum smear or positive culture for Mycobacterium tuberculosis	Absent	Present	Absent	Variable	Variable	Variable
Site of infection	Infection primarily in the lungs or other sites	Not applicable	Pulmonary	Not applicable	Extra-pulmonary	Variable	Not applicable
Progression to active TB	Likely progression to active tuberculosis disease	Unlikely	Likely	Possible	Possible	Possible	Possible

*Control* normal control, *PTB* pulmonary tuberculosis, *LTBI* latent tuberculosis infection, *EPTB* extrapulmonary tuberculosis, *OD* organ-specific tuberculosis, *Subclinical* subclinical tuberculosis.

Subsequent differential expression analysis revealed over 2000 differences among C1, C2, C3 (Fig. 2F). Particularly, there was substantial overlap in differential genes between C2 and C1, suggesting similarity in PTB development. Conversely, C3 demonstrated less overlap with C1 and C2, implying significant distinctions. This trend was further confirmed by examining the top 20 highly expressed genes, with C3 displaying pronounced differences compared to C1 and C2 (Fig. 2G). Noteworthy genes such as *IFIT3*, *TAPBP*, and *CSF3R* play pivotal roles in the host immune response to TB, exhibiting expression patterns consistent with the immunological validation trends observed in all three subgroups (Fig. 2H). For instance, *IFIT3* participates in interferon-induced antiviral immune responses, impacting host clearance of tuberculosis and inflammation regulation^25^. *TAPBP* is involved in antigen processing and presentation, influencing the immune response and antimicrobial capability in TB patients^26^. Meanwhile, *CSF3R* regulates bone marrow hematopoiesis and immune cell proliferation and differentiation, potentially affecting the host's ability to clear tuberculosis^27^.

Functional analysis further revealed associations of C1 with ATP synthesis coupled electron transport and oxidative phosphorylation pathways, C2 with receptor degradation processes, and metabolism-related amino sugar and nucleotide sugar metabolism, and C3 with cytokine-mediated signaling pathways related to anti-inflammatory processes (Figs. 2I and J; Tables S4 and S5). These findings underscore that C1, C2, and C3 exhibit distinct molecular features and functional differences, reflecting diverse immunological subtypes in tuberculosis pathology.

### Characterizing cellular compositions and immune pathway activity across PTB subgroups

**Figure 3:**
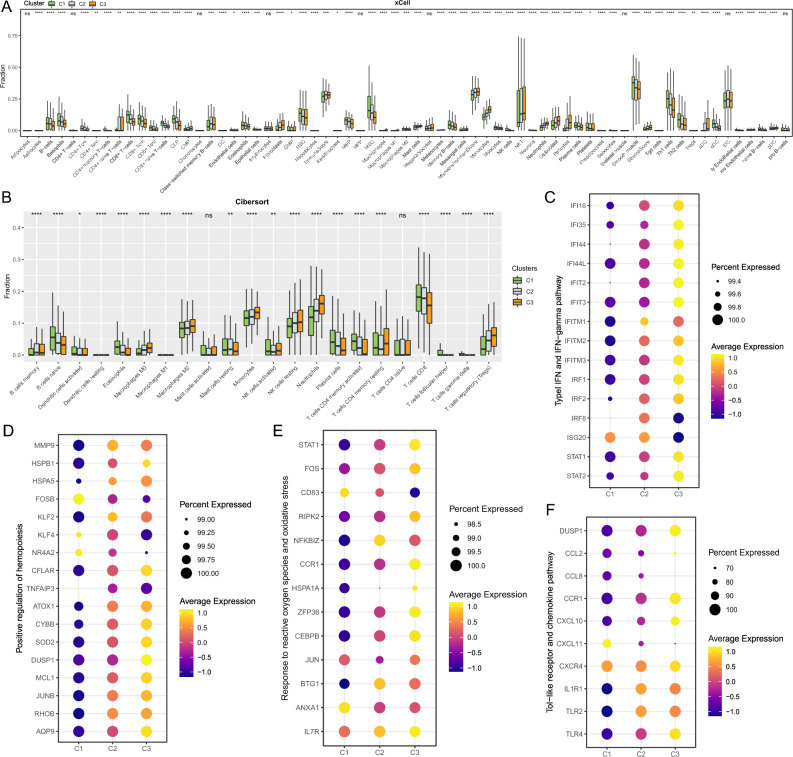
Distribution of cell infiltration and expression patterns of gene sets related to PTB progression across Fges-derived subgroups. (**A**) Boxplot illustrating the distribution of cell infiltration estimated by the xCell tool across PTB subgroups. Not significant (ns): *p* > 0.05; *p < 0.01; **p < 0.001; ***p < 0.0001; ****p < 0.00001. *P*-values were obtained through *t*-test. (**B**) Boxplot displaying the infiltration of 22 immune cell types in PTB subgroups, as estimated by CIBERSORT. Not significant (ns): *p* > 0.05; *p < 0.01; **p < 0.001; ***p < 0.0001; ****p < 0.00001. *P*-values were obtained through *t*-test. (**C**) Bubble plot depicting the expression of Type I IFN and IFN-gamma pathway-related genes among PTB subgroups. The size of each point represents the percentage of samples expressing that gene, while the color reflects the variation in gene expression among the subgroups. (**D**) Bubble plot illustrating the expression of genes related to the positive regulation of hemopoiesis among PTB subgroups. The size of each point indicates the percentage of samples expressing that gene, and the color denotes the variation in gene expression across clusters. (**E**) Bubble plot demonstrating the expression of genes related to the response to reactive oxygen species and oxidative stress among PTB subgroups. The size of each point corresponds to the percentage of samples expressing that gene, while the color represents the variation in gene expression across clusters. (**F**) Bubble plot displaying the expression of Toll-like receptor and chemokine pathway-related genes among PTB subgroups. The size of each point indicates the percentage of samples expressing that gene, and the color signifies the variation in gene expression among the subgroups.

Utilizing the CIBERSORT^20^ and xCell^19^ deconvolution tools, we conducted an extensive analysis of cell infiltration patterns in three distinct PTB subgroups. Noteworthy discrepancies were observed across the majority of cell types (55 out of 64 for xCell; 20 out of 22 for CIBERSORT) among these subgroups (Fig. 3A and B). Particularly intriguing was the consistent elevation of CD8 + T cell abundance in C1/C2 compared to C3, a trend consistently identified in both xCell and CIBERSORT results. Additionally, C3 showed the highest proportions of myeloid cells, including monocytes and macrophages. All these suggest that during the early stages of PTB (subgroup C3; Fig. 2E), patients have a high load of active Mycobacterium tuberculosis, leading to increased infiltration of innate immune cells like myeloid cells. Conversely, in the recovery stages (subgroups C1/C2; Fig. 2E), with lower bacterial levels, the immune system favors CD8 + T cells, which are important for clearing pathogens and building immunity, resulting in higher levels of these cells.

Further meticulous examination revealed a pronounced upregulation of genes associated with the Type I IFN and IFN-*γ* pathways in C3 relative to C1 and C2 (Fig. 3C). These pivotal pathways play a pivotal role in orchestrating the immune response to PTB infection, potentially fostering robust immunity against pathogens while concurrently posing the risk of immune exhaustion and tissue damage^28,29^. The robust activation of the positive regulation of hemopoiesis pathway in C3 hints at a tightly intertwined interaction between the body's immune system and hematopoietic system during PTB infection (Fig. 3D). The increased activity observed in C3 suggests an augmented demand for blood cells throughout the disease process, driven by heightened immune responses and inflammation, which potentially leads to escalated blood cell consumption and demand^30^. Additionally, the closely intertwined relationship between reactive oxygen species (ROS) and oxidative stress pathways with macrophages in tuberculosis underscores the pivotal role of these pathways in combating PTB infection (Fig. 3E)^31^. Macrophages, as pivotal constituents of the immune system, execute vital functions such as phagocytosis and pathogen elimination^32^, consistent with the highest relative proportion estimated by CIBERSORT in C3 (Fig. 3B). The diminished activity observed in PTB subgroups (C1 and C2) potentially hints at a less aggressive disease progression or the implementation of more efficacious immune regulation mechanisms. Additionally, the active involvement of Toll-like receptor (TLR) and chemokine pathways in C3 underscores their crucial role in recognizing and responding to PTB, while also orchestrating the migration and aggregation of immune cells (Fig. 3F)^33,34^.

Taken together, the heightened activity exhibited by these pathways in the C3 subtype serves to enhance the interaction between immune cells and pathogens, thereby expediting pathogen phagocytosis and clearance.

### Biomarker development for predicting PTB progression based on neural network model

**Figure 4:**
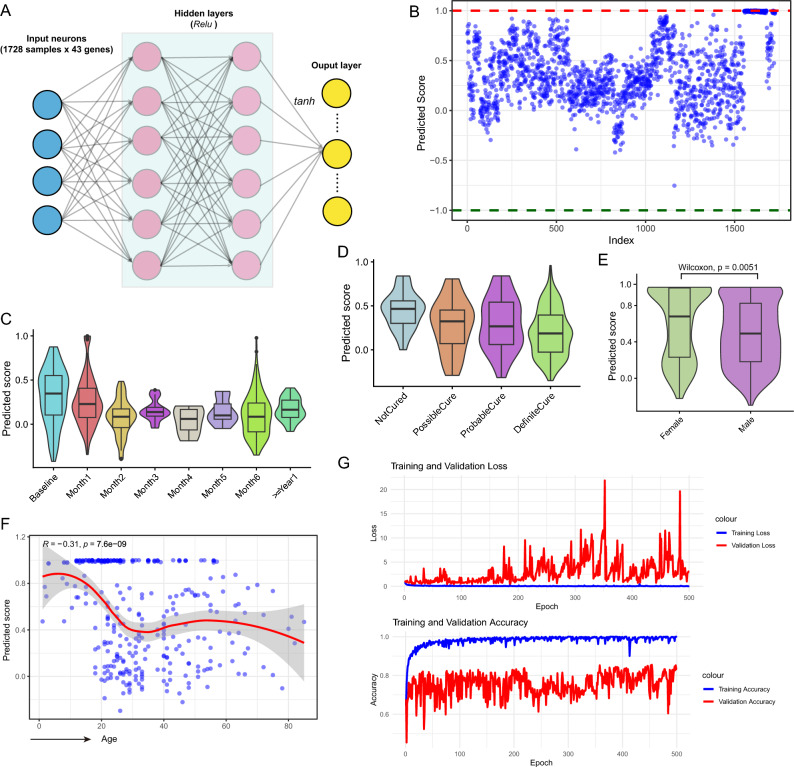
Predicted scores inferred by neural network-based model for PTB patients. (**A**) Schematic of the neural network used to assess the risk of PTB patients, comprising an input layer, two hidden layers, and one output layer (details in “Materials and methods”). (**B**) Distribution plot displaying the predicted scores generated by the neural network model. Each point represents an individual PTB patient, with scores ranging from − 1 to 1. (**C**) Violin plot combined with box plot illustrating the predicted scores at various time intervals relative to PTB exposure. The median value of predicted scores is denoted by the black line within the box. The baseline denotes the time of PTB exposure. (**D**) Distribution of predicted scores across different statuses of PTB patients. (**E**) Comparison of predicted score distributions between male and female PTB patients. *P*-values were obtained via Wilcoxon test. (**F**) Scatter plot depicting the relationship between predicted scores and ages of PTB patients. Each point represents one patient, with the blue line indicating the curve of association between age and predicted score. Pearson correlation coefficient (*R*) and associated *p*-values were calculated using a *t*-test. (**G**) Loss and accuracy metrics across epochs for training and validation sets in constructing the neural network-based model. Blue lines represent training data, while red lines represent validation data.

Considering the strong correlation between the C3 subgroup in PTB patients and the early onset and progression of the disease, an effort was made to construct a biomarker capable of predicting PTB patient progression. To achieve this, a set of 43 genes highly consistent with trends in C1, C2, and C3 within C3 was utilized as features, and a neural network model was employed to build a scoring system for PTB patients (Fig. 4A; Table S6). This model comprises two hidden layers, with the output layer utilizing a *tanh* activation function to ensure scores fall within the range of [-1, 1] (Figs. 4A and B; see “Materials and methods”). Based on model scores, the distribution at various time points relative to PTB onset was observed, revealing the highest scores at baseline, gradually declining over time, closely correlating with heightened immune-inflammatory activity in the early stages of PTB (Fig. 4C). Additionally, exploration of the distribution of predicted scores under different treatment statuses showed the highest average scores in the *NotCured* group, followed by the *PossibleCure* and *ProbableCure* groups, and the lowest in the *DefiniteCure* group, indicating better pathogen clearance in treated patients (Fig. 4D). Further analysis of predicted score distribution across genders revealed higher scores in females than males, possibly linked to biological differences (Fig. 4E). This indicates that female immune systems might display distinct response profiles, potentially posing a heightened likelihood of progressing to more severe disease stages compared to their male counterparts upon infection. Analysis also revealed a negative correlation between predicted scores and age, with younger ages (< 10 years) corresponding to higher predicted scores (Fig. 4F). The immune systems of young children may not be fully developed, making them more susceptible to TB pathogens and exhibiting more severe symptoms. Finally, considering the potential wider application of PTB subtyping inferred, adjustments were made to the feature variables of the neural network model (i.e. significant differentially expressed genes for each subtype), and the output layer was modified for classification prediction, achieving an accuracy of 92.36% on a reserved 30% validation dataset (Fig. 4G; see “Materials and methods”). This offers broader possibilities for the extension of PTB subtyping based on Cytokine signals.

### Single-cell analysis reveals enhanced MHC signaling promotes the rapid clearance of early pathogens in PTB C3 subgroup

**Figure 5:**
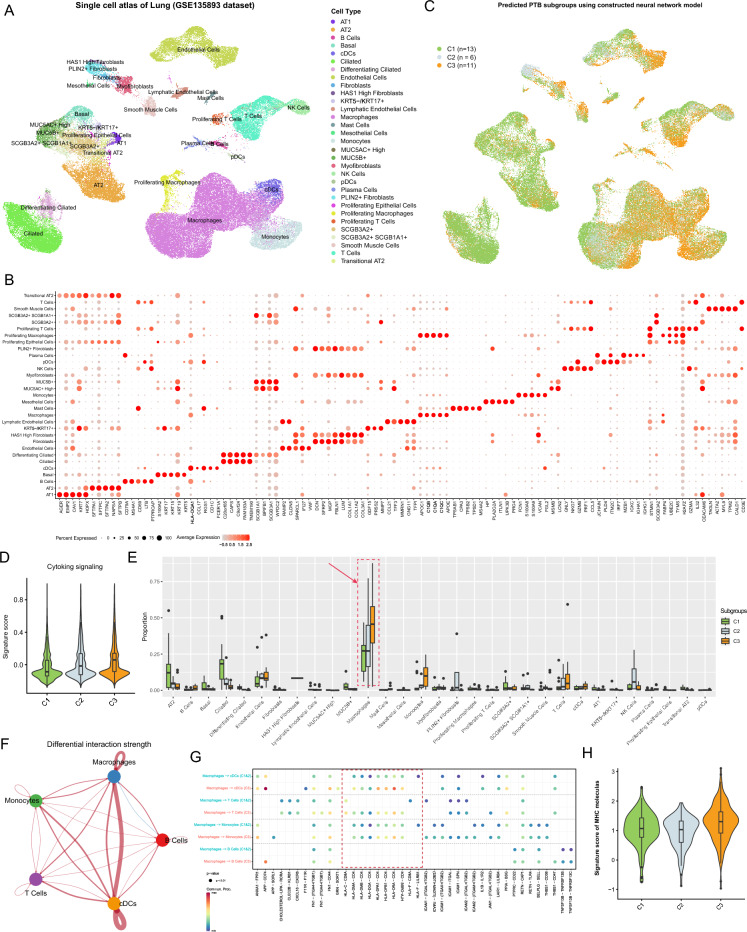
Utilizing single-cell investigation to unravel the intrinsic mechanisms underlying the three subgroups of PTB patients. (**A**) Uniform manifold approximation and projection (UMAP) visualization depicting the single-cell atlas of lung tissue from patients with fibrotic pulmonary tuberculosis. Different colors represent annotated cell types, with annotation information derived from corresponding literature reports; points represent individual cells. (**B**) Bubble plots display the average expression and positive expression proportion of the top five signature genes expressed in each cell type. Point color indicates expression level, and point size represents the percentage of expression of the gene in a specific cell type. (**C**) UMAP visualization displays the distribution of cell types in three different subtypes of PTB predicted by a neural network model. (**D**) Violin plots show the distribution of Cytokine signaling signature scores inferred from single-cell samples in different subgroups. (**E**) Box plots display the distribution of different cell types across the three predicted subtypes of PTB in single-cell samples; the black line within the box represents the median cell proportion, and points outside the box represent outlier samples. (**F**) Network diagrams illustrate the interactions between different cell types; the thickness of the lines indicates the strength of interaction between cell types. Red indicates enhancement of C3 relative to C1 & 2, while blue indicates weaker interaction. (**G**) Bubble plots demonstrate significant ligand-receptor interactions between Macrophages and B cells, T Cells, Monocytes, and cDCs. Points represent significant ligand-receptor interactions, with point color indicating the significance level of the interaction. (**H**) Violin plots depict the signature score of MHC molecules among three distinct subgroups of PTB.

Due to the current lack of sufficient available single-cell PTB data, HABERMANN et al.^23^ released single-cell data from 10 nonfibrotic control and 20 pulmonary fibrosis (PF) lungs, along with annotation information for cell types (see “Materials and methods”). Despite PF and PTB being two different lung diseases, they may share some biological features, such as abnormal changes in lung cells. Therefore, by analyzing PF data, useful information for PTB subtypes may still be acquired. Initially, the most significant top 5 marker genes for different cell types were analyzed using a differential expression strategy, showing that most of these genes are classic markers corresponding to the cell types carried in the single-cell data, implying reliable annotation results (Fig. 5A and B). To extend PTB classification to single-cell samples, a previously trained neural network model was used to predict the single-cell data sample, resulting in C1 (n = 13), C2 (n = 6), C3 (n = 11) (Fig. 5C). Clinical information of C1, C2, and C3 was compared with PF patients, showing that 61.5% of samples in C1 came from nonfibrotic control, 33.3% in C2 came from nonfibrotic control, while 100% in C3 were PF patients. This is consistent with the clinical status trends represented by C1, C2, and C3. Furthermore, using genes related to Cytokine signaling to score single cells in PF patients, single cells from C3 showed the highest signal score, followed by C2, and C1 had the lowest (Fig. 5D). This further validates that C3 has higher inflammatory activity.

Based on the single-cell classification of PF patients, the abundance distribution of different cell types in each classification was observed, showing that Macrophages were highest in C3 and lowest in C1, with similar trends observed for B Cells, T Cells, Monocytes, and cDCs (Fig. 5E). Considering that Macrophages are one of the main phagocytic cells in the body, they can clear Mycobacterium tuberculosis by engulfing and digesting pathogens. They transform pathogens into vesicles through phagocytosis and internalization, then degrade them. Therefore, changes in the interaction of Macrophages with B Cells, T Cells, Monocytes, and cDCs in C3 and C1&2 were analyzed, showing significantly enhanced communication between Macrophages and these cell types in C3 (Fig. 5F). Furthermore, the underlying molecular mechanism of this enhanced communication was analyzed, and it was interesting to note that the signal between MHC molecules and their receptors was stronger in C3 than in C1&2 (Fig. 5G); scoring of MHC expression in single cells showed the highest score in C3 (Fig. 5H). This implies that MHC molecule expression and antigen presentation capability lead to the activation and expansion of T cells in C3, thereby rapidly strengthening the attack and clearance of PTB during the outbreak phase. To facilitate comprehension, the characteristics and discoveries of PTB subgroups were ultimately consolidated in Table 2.

**Table 2:** Characteristic/findings of three PTB subgroups.

Characteristic/findings	Subgroup C1	Subgroup C2	Subgroup C3
Association with disease progression	Gradual increase during recovery phase	Gradual increase during recovery phase	Prevalent during outbreak phase
Functional characteristics	ATP synthesis	Receptor degradation, amino sugar and nucleotide sugar metabolism	Cytokine-mediated signaling related to anti-inflammatory processes
Immune-inflammatory activity	Low	Moderate	High
Activity levels of cytokines			
Type I IFN and IFN-γ pathway activity			
Positive regulation of hemopoiesis pathway			
Toll-like receptor (TLR) and chemokine pathways			
CD8 + T cell abundance	High	Moderate	Low
Macrophage cell abundance	Low	Moderate	High
Enhanced MHC signaling	Low	Low	High

## Discussion

TB poses a significant global health challenge, necessitating a thorough understanding of its immunological dynamics and clinical manifestations^7,8^. In this study, we conducted a comprehensive analysis to elucidate the complex interplay between TB and the host immune system, with a focus on identifying distinct clinical subtypes within PTB patients. PTB pathogenesis is intricately linked to the immune microenvironment, where Mycobacterium tuberculosis modulates host immune responses to establish infection.

By analyzing transcriptomic data from multiple TB cohorts, we revealed a nuanced immune gene expression profile characterized by both consistency and heterogeneity across TB patients. Notably, cytokine signaling pathways exhibited significant variability, highlighting the diverse immune profiles among TB patients. Building upon these findings, we sought to delineate distinct clinical subtypes within PTB patients. Based on the immune-related pathways and cytokine signaling activity, hierarchical clustering analysis identified three discrete PTB subgroups: C1, C2, and C3. These subtypes displayed differential immune-inflammatory activity, with C3 demonstrating the highest cytokine signaling and immune reactivity. Further molecular and functional characterization unveiled unique pathogenic mechanisms associated with each subtype. Deconvolution analysis of cellular compositions revealed significant differences among PTB subtypes, particularly in CD8 + T cell abundance. Functional pathway analysis elucidated distinct molecular profiles, with each subtype associated with specific biological processes. Notably, the robust activation of Type I IFN and IFN-γ pathways in C3 underscored its heightened immune response and potential implications for disease progression.

Driven by the associations between PTB subtypes and disease progression, we developed a predictive biomarker using neural network modeling. This biomarker demonstrated promising accuracy in predicting disease progression and treatment response, offering potential utility in guiding personalized treatment strategies for PTB patients. While existing literatures^35–38^ commonly highlight male susceptibility to PTB, the observed higher risk of severe disease progression among females, despite infection in both sexes, underscores the complexity of gender-specific immune responses to TB infection and merits further investigation. Expanding our analysis to single-cell data, we leveraged insights from pulmonary fibrosis (PF) patients to infer potential subtypes in PTB. Single-cell classification revealed distinct immune profiles corresponding to PTB subtypes, with enhanced MHC signaling identified in C3, indicative of rapid pathogen clearance during the early stages of infection. Our study provides a comprehensive understanding of TB immunopathogenesis and clinical heterogeneity, offering insights into disease progression and treatment response. By integrating multi-omics data and advanced analytical techniques, we unveil the intricate interplay between PTB and the host immune system, paving the way for personalized treatment strategies tailored to individual patient subtypes. These findings hold promise for enhancing PTB management and control efforts on a global scale. Notably, in clinical practice, it is imperative to comprehensively evaluate the overall condition of patients rather than solely focusing on the disease itself. Factors such as genetic susceptibility, comorbid symptoms, nutritional status, and psychological state can all alter a patient's prognosis and response to treatment^39–41^. Therefore, when assessing disease trends and selecting appropriate treatment strategies, it is essential to consider a combination of classification information, pathogen characteristics, and these "comorbid" factors unique to each patient.

Overall, while our study has yielded promising outcomes, it is crucial to acknowledge the inherent challenges in predicting disease trajectory based on immune-inflammatory activity in clinical practice. Firstly, immune inflammation, although a natural response to disease, exhibits significant variability in intensity and pattern among individuals. Secondly, changes in immune-inflammatory activity often lag behind other disease indicators, presenting obstacles to timely assessment. Thirdly, disease progression is influenced by a multitude of physiological and pathological factors, making it difficult for any singular immune-inflammatory marker to comprehensively capture the intricacies of the situation. Moreover, different subtypes and stages may manifest distinct patterns of immune-inflammatory responses, further complicating prediction efforts. Nevertheless, immune-inflammatory reactions remain essential as the body's primary defense against diseases, with alterations in immune cells and cytokines reflecting the body's complex interactions with pathogens and influencing disease control or exacerbation. Despite these challenges, comprehensive immune molecule analysis for subtype investigation and employing predictive modeling to forecast the disease trajectory of PTB patients can still provide valuable insights for personalized assessment of disease progression risk.

### Supplementary Information

Supplementary Information.

## Data Availability

Transcriptomic data and pertinent clinical information for the tuberculosis cohorts were obtained from publicly available databases, as detailed in Tables S1 and S2, also mentioned in the “Materials and methods” section. LL authored and reviewed all the code, which is available upon request from the corresponding author.
