# Supplementary material for: Multi-cohort analysis reveals immune subtypes and predictive biomarkers in tuberculosis
Source: Sci Rep. 2024 Jun 10;14:13345. doi: 10.1038/s41598-024-63365-5 (PMC11164950; doi:10.1038/s41598-024-63365-5)
Supplement: Supplementary file 1 — Supplementary Information. [file 41598_2024_63365_MOESM1_ESM.zip › Supplementary materials.docx]

**Supplementary materials**

**Supplementary Tables**

**Table S1**: Overview of Tuberculosis Samples.

**Table S2**: List of Knowledge-Based Functional Gene Expression Signatures (Fges).

**Table S3**: Immune-related gene set score for inferring PTB subgroups.

**Table S4**: Gene Ontology Enrichment Results of PTB subgroups.

**Table S5**: KEGG Enrichment Results of PTB subgroups.

**Table S6**: A profile matrix composed of features utilized for training neural network model.
